# Oleoyl Coenzyme A Regulates Interaction of Transcriptional Regulator RaaS (Rv1219c) with DNA in Mycobacteria*

**DOI:** 10.1074/jbc.M114.577338

**Published:** 2014-07-10

**Authors:** Obolbek Turapov, Simon J. Waddell, Bernard Burke, Sarah Glenn, Asel A. Sarybaeva, Griselda Tudo, Gilles Labesse, Danielle I. Young, Michael Young, Peter W. Andrew, Philip D. Butcher, Martin Cohen-Gonsaud, Galina V. Mukamolova

**Affiliations:** From the ‡ Department of Infection, Immunity and Inflammation, University of Leicester, Leicester LE1 9HN, United Kingdom,; the § Brighton and Sussex Medical School, University of Sussex, Brighton BN1 9PX, United Kingdom,; the ¶ Medical Microbiology, Centre for Infection and Immunity, Division of Clinical Sciences, St. George's University of London, London SW17 0RE, United Kingdom,; the ‖ Centre de Biochimie Structurale, CNRS UMR 5048, 34090 Montpellier, France,; ** INSERM U1054, Universités Montpellier I and II, 34090 Montpellier, France, and; the ‡‡ Institute of Biological, Environmental and Rural Sciences, Aberystwyth University, Aberystwyth SY23 3DD, United Kingdom

**Keywords:** ABC Transporter, Antibiotics, Fatty Acid, Ligand-binding Protein, Mycobacteria, Transcription Repressor

## Abstract

We have recently shown that RaaS (regulator of antimicrobial-assisted survival), encoded by *Rv1219c* in *Mycobacterium tuberculosis* and by *bcg_1279c* in *Mycobacterium bovis* bacillus Calmette-Guérin, plays an important role in mycobacterial survival in prolonged stationary phase and during murine infection. Here, we demonstrate that long chain acyl-CoA derivatives (oleoyl-CoA and, to lesser extent, palmitoyl-CoA) modulate RaaS binding to DNA and expression of the downstream genes that encode ATP-dependent efflux pumps. Moreover, exogenously added oleic acid influences RaaS-mediated mycobacterial improvement of survival and expression of the RaaS regulon. Our data suggest that long chain acyl-CoA derivatives serve as biological indicators of the bacterial metabolic state. Dysregulation of efflux pumps can be used to eliminate non-growing mycobacteria.

## Teaser

**Background:** RaaS mediates mycobacterial survival in nonpermissive growth conditions by controlling expression of ATP-dependent efflux pumps.

**Results:** Oleoyl-CoA regulates binding of the RaaS transcription factor to DNA and thus expression of the RaaS regulon and RaaS-mediated persistence.

**Conclusion:** The activity of bacterial efflux is regulated by metabolites that are produced during active growth.

**Significance:** Dysregulation of efflux pumps results in killing of persisting mycobacteria with low metabolic activity.

## Introduction

Tuberculosis remains a major infectious disease, with 8.6 million new cases estimated in 2012 claiming 1.3 million lives (1). Moreover, the causative agent of tuberculosis, *Mycobacterium tuberculosis*, is believed to latently infect one-third of the world's population (2). Treatment of tuberculosis requires the administration of a combination of four drugs (isoniazid, rifampin, ethambutol, and pyrazinamide) for at least 6 months. The remarkable ability of *M. tuberculosis* to survive in the infected host for years is well documented and involves the activation of complex regulatory pathways, as well as the production of specialized enzymes and transcriptional regulators (3, 4).

We have recently demonstrated that treatment of mycobacteria with antimicrobial agents targeting cell wall biosynthesis (cerulenin, isoniazid, or ethambutol) improves bacterial survival in nonpermissive growth conditions (5). This phenomenon is mediated by the transcriptional regulator RaaS (regulator of antimicrobial-assisted survival), which controls expression of putative ATP-dependent efflux pumps (Bcg_1278c/Bcg_1277 in *Mycobacterium bovis* bacillus Calmette-Guérin (BCG)^4^ and Rv1218c/Rv1217c in *M. tuberculosis*). Deletion of *raaS* from *M. tuberculosis* or *M. bovis* BCG genomes has no effect on mycobacterial growth in logarithmic phase. However, the deletion mutants in both mycobacteria are impaired in long-term survival at stationary phase. Moreover, the *M. bovis* BCG deletion mutant displays a survival defect during macrophage infection in murine lungs and spleen, whereas the *M. tuberculosis* deletion mutant does not persist during macrophage infection (5). These results demonstrate that RaaS controls mycobacterial survival in various models of *M. tuberculosis* disease. In the present study, we conducted bioinformatic analyses and predicted that fatty acid derivatives of CoA are putative ligands of RaaS. We confirmed this experimentally by demonstrating specific binding of oleoyl-CoA to RaaS and the role of oleic acid, a precursor of oleoyl-CoA, in RaaS-mediated mycobacterial survival. We propose that fatty acid metabolites produced during active growth are also involved in controlling expression of genes encoding mycobacterial efflux pumps.

## EXPERIMENTAL PROCEDURES

### 

#### 

##### Organisms and Media

The *M. bovis* BCG Glaxo strain was grown in Sauton's liquid medium supplemented with albumin-dextrose complex as described previously (5). Ethambutol and oleic acid were added, using a 1-ml syringe fitted with a 25-gauge needle, 30 days after inoculation at final concentrations of 98 and 200 μm, respectively. An equivalent volume of sterile water was added to control cultures. Bacterial viability was assayed by counting colony-forming units on 7H10 agar.

##### Transcriptional Profiling

Total RNA was isolated from 30 ml of early logarithmic (7 days) or stationary phase (31 days) *M. bovis* BCG cultures after a 24-h exposure to 200 μm oleic acid using the guanidinium thiocyanate/TRIzol method (6). DNA contamination was removed with Turbo DNA-free DNase (Ambion) before cDNA was generated using SuperScript reverse transcriptase II (Invitrogen) as described previously (7). Quantitative PCR was performed in a Corbett Rotor-Gene 6000 real-time thermocycler (Qiagen) by application of ABsolute qPCR SYBR Green mixture (Thermo Scientific) and gene-specific primers (see Table 1). Calibration curves were generated for each gene using genomic DNA (regression value of ≥0.95 and efficiency of ≥2.0). Copy numbers of specific transcripts per 1 μg of RNA were estimated using the Corbett Rotor-Gene 6000 software and normalized to 16 S rRNA expression (7). Relative gene expression in treated samples was calculated as the ratio of normalized gene copy number after treatment to normalized gene copy number before treatment and expressed as -fold change.

##### Purification of Recombinant RaaS

The *M. tuberculosis raaS* gene was cloned into the NdeI and NheI sites of the pET15-Tev plasmid to generate a hexahistidine-tagged recombinant protein (4). Protein expression in *Escherichia coli* BL21(DE3) was induced by isopropyl β-d-thiogalactopyranoside at a final concentration of 0.2 mm. Recombinant RaaS was purified using a HiTrap 1-ml IMAC HP column (Amersham Biosciences). Site-directed mutants of RaaS were generated using a GeneArt mutagenesis kit (Invitrogen) according to the manufacturer's instructions.

##### Fluorescence Anisotropy

The synthetic oligonucleotides, containing the imperfect direct repeats (Pr14F and Pr14R in Table 1), were covalently labeled with ATTO 647N succinimidyl ester dye (Invitrogen). Steady-state fluorescence anisotropy binding titrations were carried out on a Tecan Safire^2^ microplate reader using a 635-nm light-emitting diode for excitation and a monochromator set at 680 nm (bandwidth of 20 nm) for emission in buffer containing 50 mm Tris-HCl (pH 8.5) and 150 mm NaCl.

##### Isothermal Titration Calorimetry (ITC)

RaaS protein and oleoyl-CoA (Sigma) were diluted in 50 mm Tris-HCl (pH 8.5) and 150 mm NaCl. RaaS (15 μm in a 1.4-ml cell) was then titrated at 25 °C by 5-μl injections of the ligand (250 μm in the syringe) using a VP-ITC calorimeter (MicroCal). Raw data were normalized and corrected for heats of dilution of the ligand. Binding stoichiometries, enthalpy values, and equilibrium dissociation constants were determined by fitting the corrected data to a bimolecular interaction model using Origin 7 software (OriginLab).

##### Small-angle X-ray Scattering Experiments and Data Analysis

Synchrotron x-ray scattering data were collected on the SWING beamline of the SOLEIL Synchrotron (Gif-sur-Yvette, France) using a PCCD-170170 detector at a wavelength of 1.03 Å. The scattering patterns were measured by merging 10–20 data recordings with 1-s exposure time each for several solute concentrations at 1 and 3 mg/ml. To check for radiation damage, all successive exposures were compared, and no changes were detected. Using a sample-detector distance of 1.8 m, a range of momentum transfer of 0.0065 < *s* < 0.6 Å^−1^ was covered (*s* = 4πsin(θ)/λ, where 2θ is the scattering angle, and λ = 1.5 Å is the x-ray wavelength). The data were processed using standard procedures and extrapolated to infinite dilution using the program PRIMUS (8). The forward scattering, *I*(0), and the radius of gyration, *R*_g_, were evaluated using Guinier approximation, assuming that at very small angles (*s* < 1.3/*R*_g_), the intensity is represented as *I*(*s*) = *I*(0)exp(−*s*^2^*R*_g_^2^)/3).

##### EMSA

Annealed Pr14F and Pr14R (Table 1), containing the RaaS-binding site or the *raaS* upstream region (174 bp), were used for EMSAs as described previously (5). Briefly, the reaction buffer (10 μl) contained 50 mm Tris HCl (pH 8.0), 1 mm EDTA, 50 mm NaCl, 4% (v/v) glycerol, and 1 g of sheared salmon sperm DNA per reaction. ^32^P-Labeled annealed oligonucleotides and purified recombinant RaaS were added to the reaction at concentrations of 60 and 215 nm, respectively, except where stated otherwise. Oleoyl-CoA, palmitoyl-CoA, and dodecyl-CoA were added to samples as indicated. The samples were incubated for 10 min at room temperature before running on a polyacrylamide gel as described above. Following electrophoresis, gels were fixed in 20% (v/v) methanol and 10% (v/v) glacial acetic acid for 10 min, dried at 65 °C under vacuum, exposed overnight, and scanned with a Canon 5600F scanner.

**TABLE 1:** **Oligonucleotides used in this study**

Name	Sequence (5′–3′)	Use
1219PrF3	GCATGATCTAGAATTCGGCGAGCAGACGCA	EMSA
1219PrR1	TCAATGGGATCCGTTCAGGATATTAAACGT	EMSA
1219Pr14F	GGGATGAACGTACGTTTAATATCCTGAACATGCGTTCAG	EMSA
1219Pr14R	GGGCTGAACGCATGTTCAGGATATTAAACGTACGTTCAT	EMSA
RT1217F	TGTACATCGCCAGCGTCGAAA	qRT-PCR*^a^*
RT1217R	AAACATCCCGGCTTTCCAGATTCC	qRT-PCR
RT1218F	AAGACCGTCGAAAGCGGTTCACTA	qRT-PCR
RT1218R	TGAGTTCTCTCAGGCTTTCGCTGT	qRT-PCR
RT1219F	CAGGATCAGAGAGGCGGCCATCGAA	qRT-PCR
RT1219R	ATGGTCGATGACAATGCCGCGCTC	qRT-PCR
RT-drrCF	ATTGGGTTTCCGGTTTCGACAAGG	qRT-PCR
RT-drrCR	CCTTCGACAACAACGGTTTGTGCT	qRT-PCR
Myco16SF	GAAACTGGGTCTAATACCG	qRT-PCR
Myco16SR	ATCTCAGTCCCAGTGTGG	qRT-PCR

*^a^* qRT-PCR, quantitative RT-PCR.

##### Native Gel Electrophoresis

Wild-type RaaS and site-directed mutants were loaded onto 10% (w/v) polyacrylamide gels and run in 0.5× Tris borate/EDTA buffer (pH 8.5) for 20 min at 230 V. Gels were stained using colloidal Coomassie Blue stain (Sigma). Oleoyl-CoA was added at concentrations of 30 and 60 μm.

## RESULTS

### 

#### 

##### Prediction of Putative RaaS Ligands Using Bioinformatic Approaches

RaaS is annotated as a member of the TetR transcriptional regulator family (pfam00440). It has a short N-terminal DNA-binding domain and a C-terminal domain comprising the effector-binding site. The latter is responsible for the conformational and dynamic changes that regulate DNA binding. Many TetR proteins control expression of multidrug efflux pump systems in which DNA binding of the repressor is regulated by the corresponding exported drug that interacts with the ligand-binding domain (9). However, we could not detect direct binding of RaaS to any of the antimicrobials (ethambutol, isoniazid, and cerulenin) that enhance mycobacterial survival (5); the ITC traces displayed identical spikes with increasing antimicrobial concentrations (up to 5 mm).

A sequence similarity search using PSI-BLAST identified RaaS as a member of the TetR family (1.4 × 10^−10^) based on the alignment of the first 60 N-terminal residues only. This search did not reveal any close homologs that might indicate the nature of the ligand regulating the RaaS activity. Close homologs (whose ligands are unknown) are found in mycobacteria, with sequence identities ranging from 99% (*Mycobacterium marinum*; 2 × 10^−118^) to 74% (*Mycobacterium avium*; 9 × 10^−101^). More distant homologs are also found in other actinobacteria, with sequence identities ranging between 56% (*Rhodococcus jostii*; 1 × 10^−70^) to 48% (*Streptomyces sulphureus*; 8 × 10^−52^).

**FIGURE 1.**
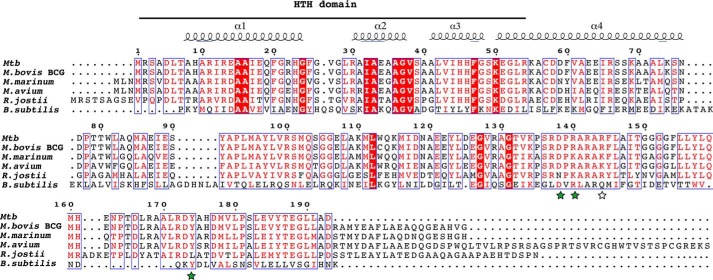
**Sequence alignment of the *B. subtilis* YsiA protein with the *M. tuberculosis* RaaS protein (Rv1219c) and its orthologs *M. tuberculosis* BCG (Bcg_1279c), *M. marinum* (WP_020726245), *M. avium* (WP_019306469), and *R. jostii* (YP_702919).** The alignment was generated using PSI-BLAST and edited. Conserved residues involved in CoA binding in *B. subtilis* YsiA are indicated by *stars*. Protein secondary element assignments, shown above the sequences, were made only for the N-terminal (DNA-binding) domain due to the low level of sequence identity within the C-terminal domain (14%). The numbering of amino acids corresponds to the *M. tuberculosis* (*Mtb*) RaaS protein. *HTH*, helix-turn-helix.

Extensive -fold recognition analysis was performed using the @TOME-2 server (10), and a structural alignment with proteins sharing low sequence identity (<20%) with RaaS was generated. These distantly related proteins share a strong hydrophobic ligand-binding pocket within the C-terminal domain. One of these proteins, YsiA (FadR), is a transcriptional regulator of the fatty acid degradation pathway in *Bacillus subtilis*, and its structure has been solved in complex with stearoyl-CoA (Protein Data Bank ID 3WHB) (11). Comparative modeling of RaaS suggested that it has features compatible with the binding of the CoA moiety of the acyl-CoA ligand, despite a low sequence identity to the regulator domain of YsiA (14%) (Fig. 1). In particular, the C-terminal domains (involved in ligand binding) of both proteins contain conservation clusters around a short motif D*X*R (Asp-138 and Arg-140 in RaaS). The conserved aspartate does not interact directly with the effector, but instead, it is involved in N-terminal cap of an α-helix and in the formation of a salt bridge with the conserved arginine. Arg-140 is then well positioned to directly interact with the adenosine acyl-CoA moiety of the YsiA ligand (ID 3WHB). We examined residues in the neighborhood position of the D*X*R motif. The Gln-154 lateral chain in FadR points toward the ligand in the YsiA structure. This residue is Arg-144 in RaaS. The substitution of glutamine to arginine suggests a possible salt bridge with the diphosphate group of acyl-CoA. Arg-144 is conserved in the close RaaS homologs and in FadR. In addition, for the RaaS models generated, Arg-144 is compatible with acyl-CoA binding. Further analysis of the dimeric structure of YsiA highlighted the possible role of Tyr-174 (corresponding to Tyr-174 in RaaS). This residue in the monomer is in contact with the ligand molecule bound to the other monomer. The aromatic ring of the tyrosine is stacked with the adenine ring of the acyl-CoA ligand. Thus, according to our dimer model, Tyr-174 in RaaS was postulated to play the same role as Tyr-174 in YsiA to stabilize the bound acyl-CoA. On the basis of these observations, we hypothesized that CoA derivatives of fatty acids might regulate RaaS-DNA binding.

##### Oleoyl-CoA Regulates RaaS Binding to DNA

**FIGURE 2.**
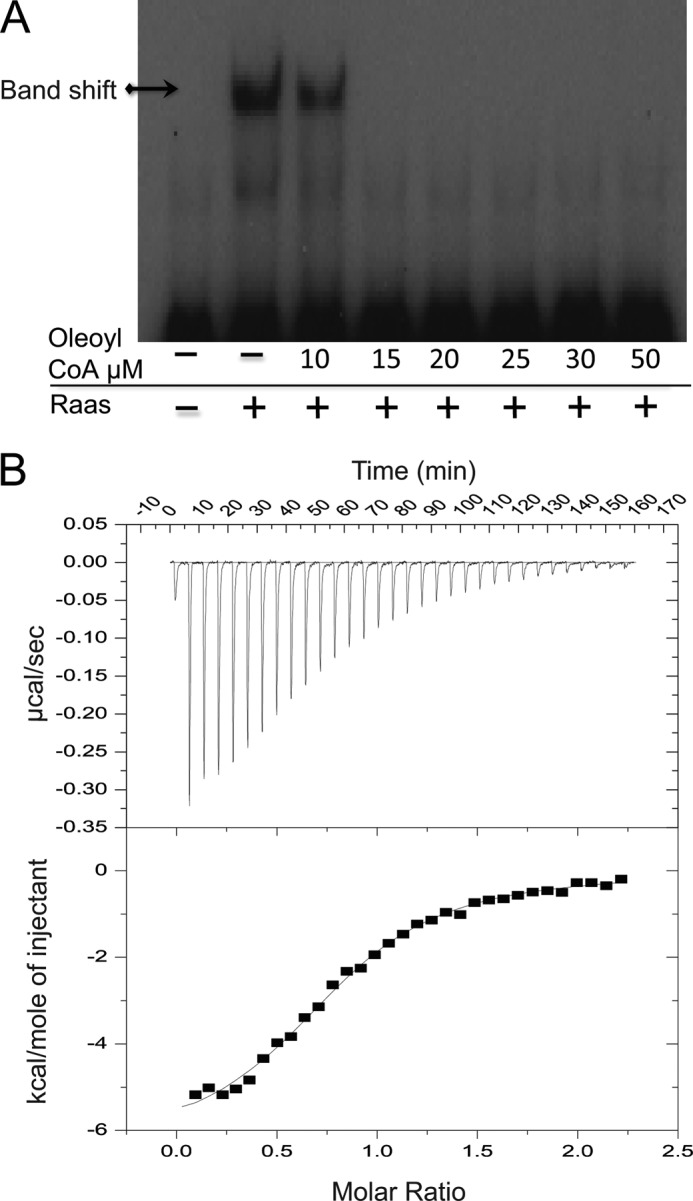
**Oleoyl-CoA regulates RaaS binding to DNA.** *A*, purified RaaS (215 nm) was mixed with radiolabeled annealed Pr14F/Pr14R oligonucleotides (60 nm); oleoyl-CoA was added as indicated. *B*, characterization of the interaction between RaaS and oleoyl-CoA by ITC. Samples were prepared as described under “Experimental Procedures.” Oleoyl-CoA bound to RaaS with a *K_d_* of 3.65 ± 0.28 μm in an enthalpy-driven reaction (Δ*H* = −6.42 ± 0.14 kcal/mol). The band shift identifying RaaS binding to DNA is marked with and *arrow*.

Mycobacterial growth media and eukaryotic cells (a natural niche for mycobacterial replication and persistence) are rich sources of oleate (12). Mycobacteria convert oleate to oleoyl-CoA by fatty acyl-CoA ligases (13), and oleoyl-CoA is an abundant fatty acid precursor for the synthesis of mycobacterial cell wall components. Therefore, we tested the effect of oleoyl-CoA on the RaaS-DNA interaction using EMSAs. As shown in Fig. 2*A*, oleoyl-CoA was able to completely abolish the RaaS-mediated band shift at concentrations ≥15 μm. Using ITC, we demonstrated direct binding of oleoyl-CoA to RaaS and a dissociation constant (*K_d_*) for oleoyl-CoA binding to RaaS of 3.65 ± 0.28 μm (Fig. 2*B*).

**FIGURE 3.**
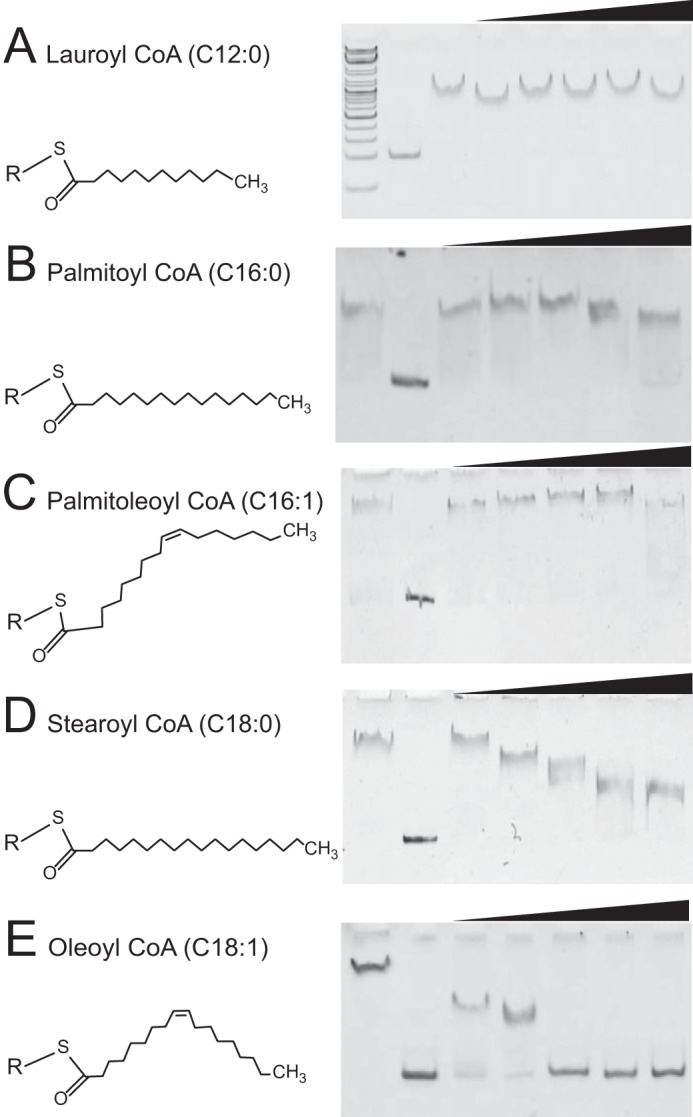
**Effect of acyl-CoA derivatives on RaaS-DNA binding.** Lauroyl-CoA (*A*), palmitoyl-CoA (*B*), palmitoleoyl-CoA (*C*), stearoyl-CoA (*D*), and oleoyl-CoA (*E*) were added at 0, 5, 10, 25, 50, and 100 μm, respectively.

We next investigated whether other acyl-coenzyme derivatives have any effect of the RaaS-DNA complex. Lauroyl-CoA (C_12:0_), palmitoyl-CoA (C_16:0_), and palmitoleoyl-CoA (C_16:1_) had no effect on the band shift in the EMSA (Fig. 3, *A–C*). Stearoyl-CoA (C_18:0_) showed a dose-dependent inhibition; however, it did not eliminate RaaS-DNA binding even at the highest concentration tested, 100 μm (Fig. 3*D*). These results suggest that RaaS preferably binds C_18_ fatty acid derivatives (C_18:1_ oleoyl-CoA or, to some extent, C_18:0_ stearoyl-CoA). Longer acyl-CoA metabolites (>C_18_) could not be tested in our assay due to their poor solubility.

##### Role of Conserved Amino Acids in DNA and Ligand Binding

Our thorough sequence structure analysis (see above) suggested that three amino acids could be responsible for acyl-CoA binding in RaaS: Arg-140, Arg-144, and Tyr-174. Using site-directed mutagenesis, we generated three RaaS mutants in which these residues were replaced with alanine: R140A, R144A, and Y174A.

We first confirmed the structural integrities of the RaaS proteins by performing small-angle x-ray scattering experiments. Guinier plot analysis yielded radii of gyration of 30.4 ± 1, 29.7 ± 1.8, and 30.7 ± 1.3 Å for the wild-type and mutant R144A and Y174A proteins, respectively. The R140A mutant was unstable at high concentrations, and therefore, it was not possible to obtain reliable data for this protein. The apparent molecular masses calculated from the *I*(0) values were 40, 44, and 40 kDa for wild-type RaaS, R144A, and Y174A, respectively, confirming that all proteins were present as folded homodimers in solution.

**FIGURE 4.**
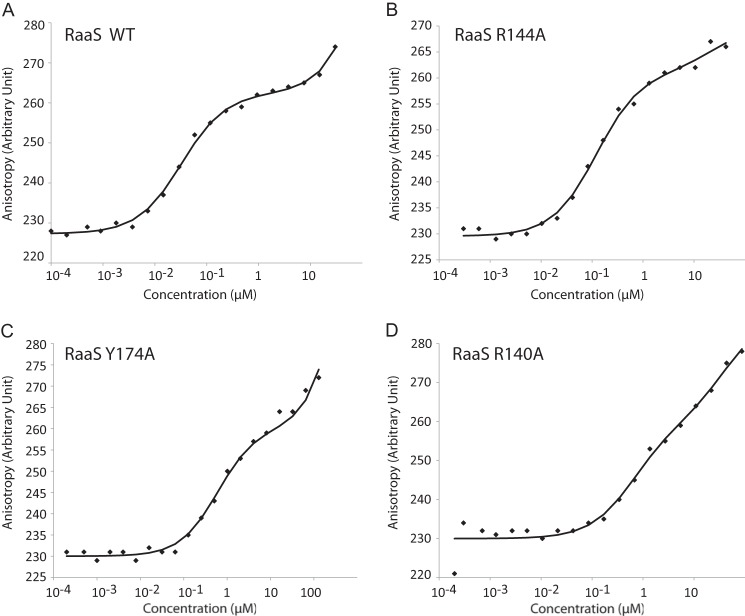
**Mutations in the predicted ligand-binding domain influence RaaS-DNA binding as measured by fluorescence anisotropy.** Shown are the anisotropy affinity profiles for the interactions of ATTO 647N-DNA (4 nm) with wild-type RaaS (*A*), R144A (*B*), Y174A (*C*), and R140A (*D*).

**FIGURE 5.**
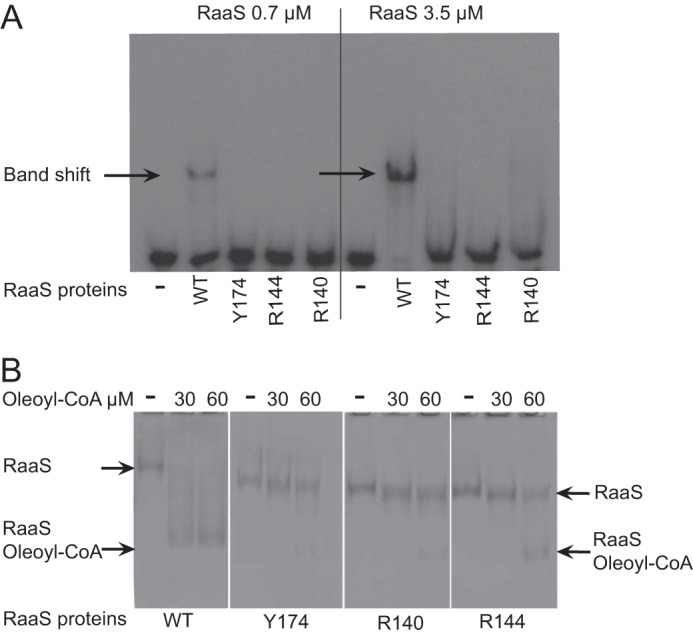
**Site-directed mutations alter RaaS interaction with DNA and ligand binding.** *A*, the mutant proteins were used for EMSA. Radiolabeled ^32^P-labeled annealed Pr14 oligonucleotides (200 nm) were mixed with purified RaaS at two concentrations (0.7 and 3.5 μm). *B*, native electrophoresis of wild-type RaaS and mutants R140A, R144A, and Y174A. RaaS and its mutants (200 nm) were loaded onto a 10% (w/v) polyacrylamide gel. *Arrows* indicate a shifted protein band corresponding to the complex between RaaS and oleoyl-CoA.

We next investigated their binding to DNA. All three mutations caused substantial increases in the dissociation constant measured by fluorescence anisotropy, ranging from 129 ± 70 nm for R144A to 611 ± 120 and 654 ± 300 nm for Y174A and R140A, respectively, compared with 31 ± 6 nm determined for wild-type RaaS (Fig. 4). The reduced DNA binding affinity resulting from these mutations was consistent with the results of EMSAs. As shown in Fig. 5*A*, the mutant proteins did not cause substantial DNA band shifts under the conditions used. The observed changes in the RaaS-DNA binding affinity in the wild-type and mutant proteins could be explained by altered RaaS conformation and/or flexibility, as observed in other TetR-like regulators (14). We therefore could not use EMSA to investigate the influence of oleoyl-CoA on the interaction of mutant RaaS with its DNA-binding site. The mutants also required higher oleoyl-CoA concentrations for titration in ITC experiments compared with wild-type RaaS. In fact, the RaaS-oleoyl-CoA binding affinities for the mutant proteins could not be measured by ITC because oleoyl-CoA aggregates at the high concentrations required (>300 μm), reducing data quality. We therefore employed native gel electrophoresis to investigate oleoyl-CoA binding to RaaS mutants, measuring the effect of oleoyl-CoA binding on migration of RaaS proteins. As shown in Fig. 5*B*, the addition of 30 or 60 μm oleoyl-CoA resulted in more rapid migration of RaaS and an apparent reduction in size (indicated with an *arrow*), which could be explained either by dissociation of the dimer to a monomeric form or by an altered protein charge or flexibility. Treatment of the mutant proteins with 30 μm oleoyl-CoA had no pronounced effect on their migration patterns, whereas the addition of 60 μm oleoyl-CoA had a partial effect, resulting in formation of two forms of RaaS (Fig. 5*B*). These results suggest that the conserved amino acids in the ligand-binding domain are indeed involved in binding oleoyl-CoA.

##### Oleic Acid Influences RaaS-mediated Biological Effects

**FIGURE 6.**
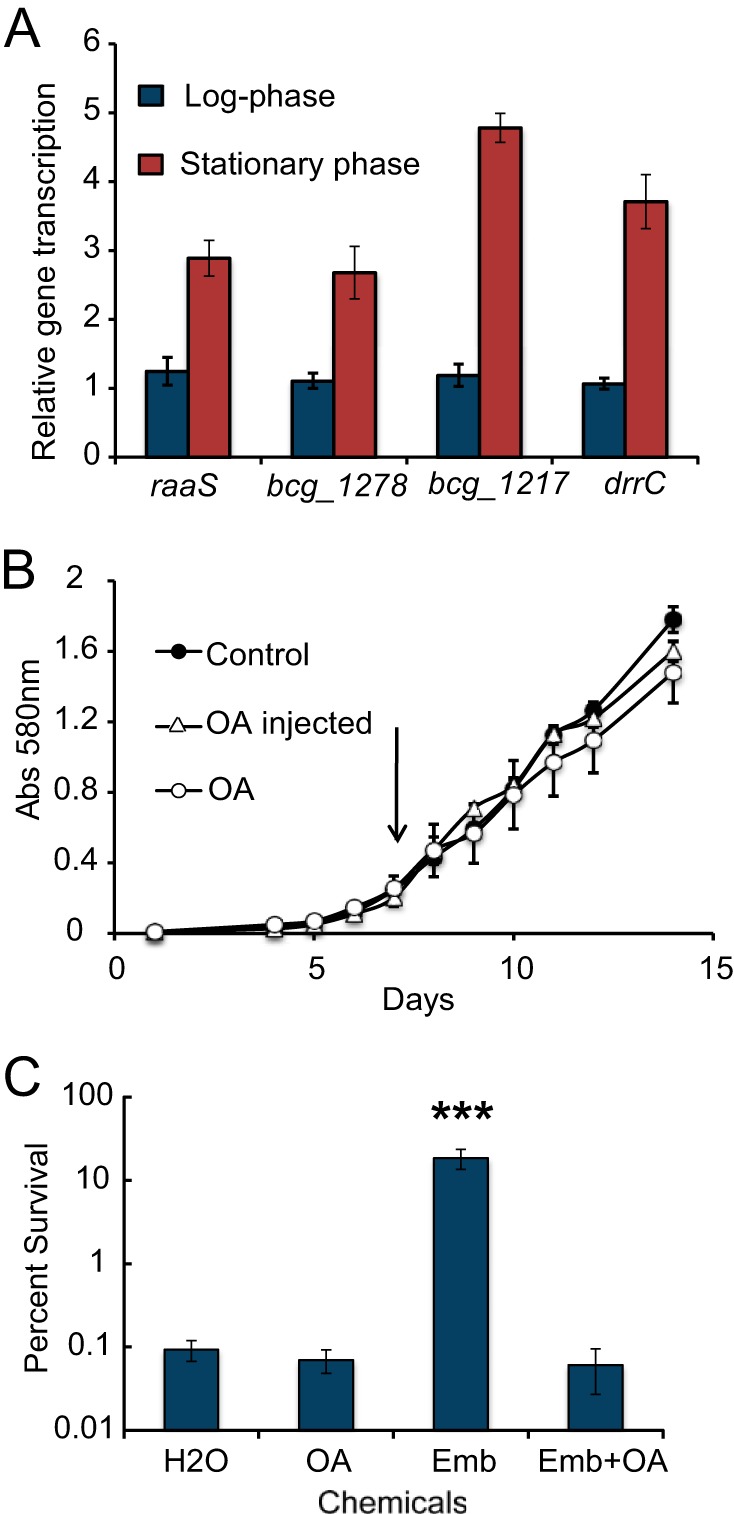
**Oleic acid influences RaaS-mediated biological phenomena.** *A*, effect of oleic acid on expression of genes from the RaaS regulon. Shown is the relative expression of selected genes from the RaaS regulon in stationary phase cultures treated with oleic acid. The genes were statistically significantly overexpressed in oleate-treated stationary cultures compared with water-treated controls (*t* test, *p* < 0.01). *B*, effect of oleic acid on the growth of *M. bovis* BCG at logarithmic phase. Oleic acid (*OA*; 200 μm) was added before inoculation of bacteria (○) or to a growing mycobacterial culture with *A*_580 nm_ = 0.2, indicated by the *arrow* (▵). *C*, oleic acid eliminates the RaaS-mediated improvement of *M. bovis* BCG survival. Oleic acid abolished the protective effect of ethambutol treatment under nonpermissive growth conditions. *Emb*, ethambutol. ***, statistically different from the water control and ethambutol + oleic acid (*t* test, *p* < 0.001).

Finally, to confirm the biological relevance of our findings with oleoyl-CoA, we investigated the effect of oleic acid on expression of the RaaS regulon and the drug-mediated improvement of *M. bovis* BCG survival. We have recently shown that RaaS is an autorepressor protein, and its regulon includes several genes encoding efflux pumps and conserved proteins of unknown function (5). In mycobacterial cells, oleic acid is converted to oleoyl-CoA by the coordinated action of fatty acyl-AMP ligases and acyl-CoA-synthesizing fatty acyl-CoA ligases (12), resulting in an increase in intracellular acyl-CoA concentration. An increase in oleoyl-CoA concentration should prevent the transcriptional repressor RaaS from binding DNA, leading to elevated expression of the RaaS efflux regulon in nonreplicating mycobacteria, which is detrimental to long-term survival. Expression levels of selected genes belonging to the RaaS regulon were measured using quantitative RT-PCR. We compared the effect of oleic acid treatment on expression of *raaS*, *bcg_1277c*, *bcg_1278c*, and *drrC* in logarithmic and stationary growth phases. Oleic acid had no statistically significant effect on expression of these genes in logarithmic phase (Fig. 6*A*). However, expression of RaaS-regulated genes (*bcg_1277c*, *bcg_1278c*, and *drrC*) was induced by 4.8-, 2.7-, and 3.7-fold, respectively, in stationary phase *M. bovis* BCG cultures 24 h after treatment with oleic acid (Fig. 6*A*). Expression of *raaS* (in the same operon as *bcg_1277c* and *bcg_1278c*) in stationary phase mycobacteria was also increased after treatment with oleic acid. Exposure of log phase mycobacteria to oleic acid did not result in growth alteration (Fig. 5*B*). Strikingly, the addition of oleic acid to 1-month-old stationary phase cultures completely abolished the antimicrobial survival-enhanced effect (Fig. 6*C*) mediated by RaaS, thus implicating oleoyl-CoA in the regulation of long-term mycobacterial survival.

## DISCUSSION

Expression of bacterial operons is tightly controlled by transcription factors, repressors or activators, which alter transcript levels in response to physiological bacterial state or environmental cues (15). In some cases, as pertinent here for RaaS, repressors are cotranscribed with their regulon (14). RaaS is a transcriptional regulator that plays an important role in long-term mycobacterial survival *in vitro* and *in vivo* (5). Moreover, RaaS mediates an improvement in mycobacterial survival after exposure of nonreplicating bacteria to antimicrobial agents targeting cell wall biosynthesis. Here, we have demonstrated that the RaaS-DNA complex is regulated by acyl-CoAs, specifically oleoyl-CoA and, to a lesser extent, stearoyl-CoA (Figs. 2*B* and 3). The addition of oleic acid, a precursor of oleoyl-CoA, completely abolished the survival-promoting effects of ethambutol and dysregulated the RaaS regulon (Fig. 5), confirming the biological relevance of oleoyl-CoA as a regulator of mycobacterial persistence.

**FIGURE 7.**
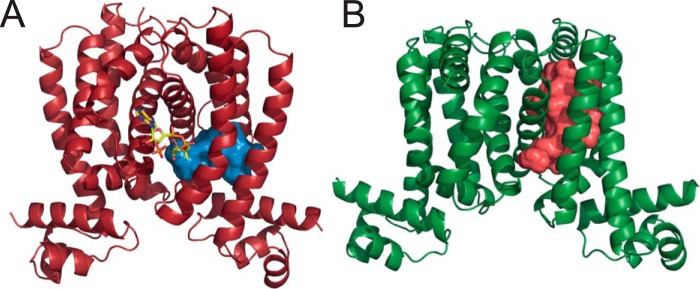
**Volume of hydrophobic cavities in RaaS and YsiA.** Shown are the crystal structures of YsiA in *red* (Protein Data Bank ID 3WHB) (*A*) and RaaS in *green* (ID 4NN1) (*B*). The two proteins display hydrophobic cavities of different volumes: 403 Å for YsiA (shown in *blue* in *A*) and 880 Å for RaaS (shown in *red* in *B*). The volumes were calculated with POCASA 1.0, and the figure was generated using PyMOL and HOLLOW (27).

During the preparation of this manuscript, a crystal structure of RaaS (Rv1219c) was solved (16). The structure analysis confirmed our predictions concerning the importance of Arg-140 and Arg-144, but not Tyr-174, in ligand binding. However, the structure revealed the presence of a very large hydrophobic cavity of 880 Å compared with the calculated volume of the aliphatic chain of oleoyl-CoA of 345 Å (Fig. 7*B*). The RaaS cavity size significantly exceeds volumes of the binding pocket of YsiA (403 Å) (Fig. 7*A*) or other mycobacterial TetR regulators such as EthR (399 Å; Protein Data Bank ID 4DW6) and KstR (190 Å; ID 3MNL). This striking difference in the RaaS structure indicates that other longer chain acyl-CoAs or complex lipids containing fatty acids might potentially fit into the binding pocket and regulate the DNA-binding activity of RaaS. Mycobacteria are able to produce and export a great variety of lipids containing fatty acids from oleic acid itself (17) to acyl-trehaloses (18). It is possible that the RaaS-regulated Rv1218c/Rv1217c pump is involved in the export of these oleate-containing lipids. Moreover, DrrC (which may also be regulated by RaaS) has been implicated in the export of the complex lipid phthiocerol dimycocerosate in *M. tuberculosis* (19).

**FIGURE 8.**
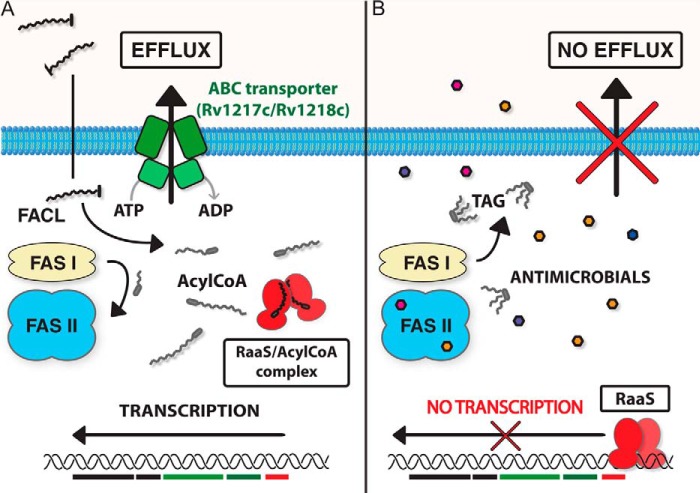
**Schematic representation of proposed RaaS function.** *A*, in growing cells, RaaS is bound by acyl-CoA, and the Rv1218c/Rv1217c-encoded ATP-dependent pump is expressed. *B*, antimicrobial treatment of stationary phase *M. bovis* BCG cells results in inhibition of residual cell wall biosynthesis and depletion of acyl-CoA. In the absence of circulating acyl-CoA, RaaS binds to its recognition sequence, preventing expression of the downstream genes encoding the ATP-binding cassette (*ABC*) transporter. *FAS*, fatty acid synthase; *FACL*, fatty acyl-CoA ligase; *TAG*, triacylglycerols. Dysregulation of this system preferentially impairs slow/non-growing bacilli.

Derivatives of fatty acids have been previously demonstrated to regulate the expression of mycobacterial lipid transporters (20). Our findings support the importance of acyl-CoA metabolites in the regulation of mycobacterial efflux and long-term persistence. As free acyl-CoA accumulates, RaaS is released from its DNA-binding site, and downstream genes encoding efflux pumps are expressed (Fig. 8*A*). Depletion of free acyl-CoA allows RaaS to bind to DNA, resulting in repression of the downstream transporter genes (Fig. 8*B*).

ATP-dependent efflux pumps are integral components of the energy and metabolic circuitry of growing cells, and their activity depends on the metabolic state of bacteria, which may be finely tuned by metabolites binding to transcriptional regulators. In non-growing cells, the levels of acyl-CoAs drop following the slowing down of metabolism. Under stressful conditions (for example, hypoxia or iron limitation), mycobacteria can redirect carbon flux from the tricarboxylic acid cycle to alternative metabolic pathways and to the synthesis of triacylglycerols (21, 22). This mechanism is important for long-term survival, as it generates storage compounds, removes toxic fatty acid derivatives, and depletes free fatty acids and their CoA derivatives (21, 22).

We hypothesize that under stressful conditions or to survive long-term during infection, free RaaS binds to its DNA recognition sequence, repressing the transcription of this cluster of efflux pump genes and itself in a classical feedback loop (Fig. 8). We further propose that antimicrobial treatment potentiates this process, directly or indirectly inhibiting fatty acid synthases and rapidly depleting residual (C_16_–C_18_ chain) fatty acid precursors (23–25).

We have shown that RaaS is a component of the complex regulatory mechanisms orchestrating a coordinated down-regulation of energy-consuming processes and the activation of long-term persistence. This is supported by our previous findings that *M. tuberculosis* employs the RaaS-mediated mechanism for *in vitro* persistence in prolonged stationary phase and during *in vivo* macrophage and mouse infections (5). Transposon inactivation of Rv1218c results in the highest fitness cost in macrophage culture (26), but the precise physiological role of the Rv1218c/Rv1217c (Bcg_1278c/Bcg_1277c) pump remains unknown. We propose that Rv1218c/Rv1217c likely transports lipid molecule(s) that are important for the initial stages of infection and may become toxic or are dispensable during nonreplicating persistence. Our findings suggest that this putative lipid contains fatty acid moieties (oleic and possibly also stearic acids), which are highly abundant in growing mycobacteria (as CoA derivatives) and are rapidly depleted in nonpermissive growth conditions. Our sequence analysis extended to Rv1216c and Rv1215c (Bcg_1276c and Bcg_1275c) highlights distant similarities to an *S*-adenosylmethionine-dependent membrane-embedded methyltransferase and a lipid esterase/transferase. Accordingly, these proteins might be predicted to modify a lipid precursor to be secreted by the Rv1218c/Rv1217c (Bcg_1278c/Bcg_1277c) efflux pump. Future experiments will focus on the nature of the molecules transported by Rv1218c/Rv1217c (Bcg_1278c/Bcg_1277c) and elucidation of their precise roles in mycobacterial growth, infection, and persistence. Our findings indicate that dysregulation of efflux pumps can be employed to kill non-growing mycobacteria.

## Footnotes

4 The abbreviations used are:

BCG: bacillus Calmette-Guérin
ITC: isothermal titration calorimetry.
